# Lasertherapy efficacy in temporomandibular disorders: control study

**DOI:** 10.1590/S1808-86942010000300004

**Published:** 2015-10-20

**Authors:** Thiago de Santana Santos, Marta Rabello Piva, Maria Helena Ribeiro, Antonio Azoubel Antunes, Auremir Rocha Melo, Emanuel Dias de Oliveira e Silva

**Affiliations:** 1 Expert in Maxillo-Facial Surgery – Brazilian College of Maxillofacial Surgery; MSc student in Maxillofacial Surgery and Traumatology – Dentistry School of Pernambuco, FOP/UPE; 2 PhD in Oral Pathology – Rio Grande do Norte University – UFRN, Professor of Oral Pathology of the Federal University of Sergipe, UFS; 3 Expert in Endodontics – Federal University of Rio de Janeiro – UFRJ, Professor of Dentistry – Federal University of Sergipe – UFS; 4 Expert in Maxillofacial Surgery and Traumatology – Dentistry School of Pernambuco – FOP/UPE., MSc Student in Maxillofacial Surgery and Traumatology – Dentistry School of Ribeirão Preto, FORB-USP; 5 Expert in Maxillofacial Surgery – Brazilian College of Maxillo-Facial Surgery; MSc student in Maxillofacial Surgery and Traumatology – Dentistry School of Pernambuco, FOP/UPE; 6 Expert in Maxillofacial Surgery and Traumatology, head of the Residency Program in Maxillofacial Surgery and Traumatology – Oswaldo Cruz University Hospital HUOC/UPE. Universidade Federal de Sergipe (UFS) Universidade de Pernambuco (UPE)

**Keywords:** lasers, temporomandibular joint dysfunction syndrome, temporomandibular joint disorders

## Abstract

Temporomandibular dysfunction is characterized by the presence of painful joint/muscular symptoms muscle in the face. The main justification for the use of lasers in laser therapy dysfunction is its analgesic effect, which was observed in most studies in the literature.

**Aim:** We evaluated the effectiveness of laser therapy in the treatment of temporomandibular disorders.

**Methods:** 50 volunteers with temporomandibular disorders were divided into two groups (control and experimental) had amplitudes of movements of mouth opening, right and left laterality recorded before and after laser application. Was also recorded, the score the individual gave to pain by visual analog scale and, through physical examination, the pain points. We used the AsGaAl laser with a 40mW power, with 80J/cm2 for 16 seconds at four selected points for just one session with reassessment after a week. Study design: Clinical.

**Results:** It was noted that laser therapy increased the mean amplitude of mandibular movements (p = 0.0317) and decreased significantly (43.6%) the pain intensity measured by the visual analog scale.

**Conclusions:** The laser decreases the painful symptoms of the patient after application through its analgesic and/or a placebo effect.

## INTRODUCTION

Temporomandibular joint dysfunction (TMJD) or temporomandibular disorder (TMD) is characterized by facial muscle/joint pain, especially in the acute phase^1^, ^2^, ^3^, ^4^, ^5^. TMD has numerous signs and symptoms: muscle and/or joint pain, joint noises, earache, mandible shifting and, in the most severe cases, dislocations^2^, ^3^.

The main justification as to the use of low intensity laser (laser therapy) on TMD is its analgesic effect reported by most of the studies found in the literature^6^, ^7^, ^8^, ^9^, ^10^, ^11^, ^12^, ^13^, ^14^, ^15^, ^16^.

According to Medeiros^13^, many studies show that laser increases the amount of collagen in the wound, causing angiogenesis and reducing lesion repair time, increasing the number of cells available for healing. According to Freitas et al.^14^, often times laser therapy can be used in lieu of anti-inflammatory medication, thus, preventing side effects. Nonetheless, Beckerman et al.^7^ reported effects arising from laser therapy – transitional pins and needles, mild erythema, a burning feeling, pain increase and exanthema.

The low intensity laser is, in many cases, a new treatment mode for the treatment of maxillofacial region disorders such as joint pain, neuralgias and paresthesias^7^.

Despite the numerous treatment modes available for TMD, only low intensity laser has proven capable of relieving pain in minutes after administration, bringing about a significant improvement to the patient. Despite all these benefits brought about by laser treatment, it is not the definitive treatment for TMD. It works as a coadjuvant in the treatment alleviating pain thanks to the laser's analgesic effect, which allows the patient to promptly resume her/ his functions, providing greater comfort6. Nonetheless, for a safe use of laser with patients, the professionals must be trained to use the equipment, thus reducing the possibility of iatrogenic effects.

The goal of the present study was to assess the efficacy of laser treatment in the management of TMD by means of the Visual Analogue Pain Scale (VAPS) and measuring the range of mandibular movements.

## MATERIALS AND METHODS

This study was made up of a descriptive survey of the clinical picture and results obtained after the exam and administration of laser treatment, with later reassessment of the laser use, in TMD patients feeling pain. We had 50 volunteers who agreed to sign the Free and Informed Consent Form (FICF). This study followed the requirements established on Resolution # 196/96 from the National Health Board/Ministry of Health – Brazil, and was approved by the Ethics in Research Committee of our Institution, under protocol # 137/2004.

The patients were broken down in two groups of 25 patients each – study and control groups. According to the methodology advocated by Kulekcioglu et al.^15^ – and with some changes done exclusively for this study we employed the VAPS so that later we would select four laser application points. These four points would be those which had the highest sensitivity/pain score among 17 assessed sites: joint capsule (lateral, posterior and superior); masseter (anterior, inferior); temporal (anterior, middle, posterior, origin and insertion); medial and lateral pterygoid, sternocleidomastoid (superior, inferior and middle); trapezius muscle (origin and superior).

After filling out the guided questionnaire and undergoing physical exam, we assessed the individual's pain intensity through the EVAD before using the laser and after one of week of use (reassessment) according to what was done in other studies^6^,^10^,^11^,^14^,^15^,^17^,^18^.

We used the Ultrablue dental laser (D.M.C. Equipment®), with a maximum power of 120 J/cm2, diode laser power of 800 mW, with beam divergence of 8°x28°, with irradiation area of 5mm2 without the application tip and an area of 4 mm2 with the administration tip, there was a 20% power loss with the 830 nm wavelength (visible in red) and AsGaAl diode laser semiconductor, it was punctually deployed on the TMJ and smaller muscles, based on shooting the laser on strategic points over the affected area and using the scanning technique, in which the laser is moved up and down throughout the entire affected area. In the control group the device was off during the application. In order to make sure the patient would not perceive it, he/she was asked to close the eyes, besides wearing goggles – as a means of eye protection. The laser therapy efficacy was also assessed through measuring the range of motion of the maximum mouth opening the patient could get, as well as left and right laterality.

We carried out a descriptive analysis with a quantitative approach of the Visual Analogue Scale (VAS) values, mouth opening amplitude, lateral movements and main places which were most affected by sensitivity/pain. We used the Epi Info 3.2 software, through which we did the ANOVA statistical tests (variance analysis) -in order to assess pain intensity; and that of Wilcoxon -in order to assess the range of mandible motion.

## RESULTS

From the study group we obtained an initial average value of the pain score, through the Visual Analogue Pain Scale, of 5.14. After the reassessment we had the mean score of 2.9. According to the ANOVA test, the result was statistically significant (p=0.0317), which was different from that of the control group, which initial mean value was 5.4 and after reassessment it was 4.25 – a statistically significant result (p=0.2371).

According to Table 1, only one patient of the group presented an increase in pain intensity during reassessment.

**Table 1:** VAPS Score at the initial moment and after reassessment

Gr Study Group	1	2	3	4	5	6	7	8	9	10	11	12	13	14	15	16	17	18	19	20	21	22	23	24	25
Initial	7,5	3,5	6	3,5	5	6	7,5	2,5	8	6	2	5	8	3	5	7	5	3,5	5	3	4	7	3	10	2,5
Final	3	3,5	3	2,5	3,5	3	5	2	3	8	2	0	5	1	0	3	3	3	3	2	2	5	2	4	1
p-Value	0.0317*																								
G G Control Group	26	27	28	29	30	31	32	33	34	35	36	37	38	39	40	41	42	43	44	45	46	47	48	49	50
Initial	6	4,5	8	3	10	5	7	7	3	6	5	3	8	6	4	3,5	9	2	4	5	3	7	4	10	2
Final	5	4	9	1	10	5	9	6	0	5,5	3	5	6	5	4	3	7	2	1	4	2,5	5	5	9	0
p-Value	0,2371*																								

* ANOVA Test

Of the studied patients, 92.0% had pain in one or more points on the Masseter muscle (origin, body and insertion) and/or in two on the TMJ (pre-auricular and intrameatal) (Table 2).

**Table 2:** Pain sites during mandibular movement in the patients of the study group

Site	%
TMJ	73,7%
Temporal	10,5%
Masseter	15,8%
Total	76,0%

**Graph 1:**
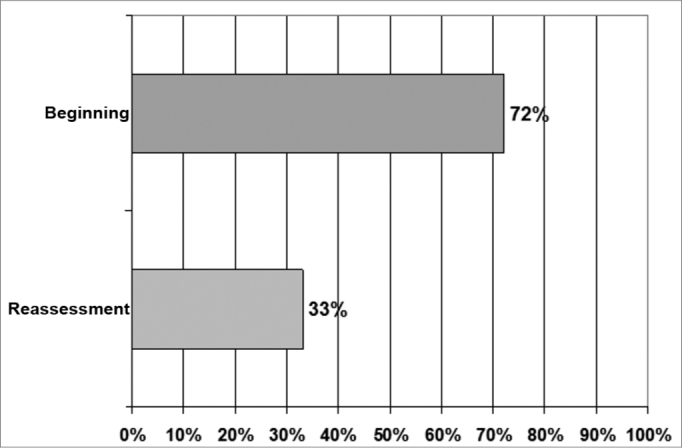
Joint noises present before and after the administration of laser therapy in the study group.

In the study group 72.0% of the patient had a mandibular cracking sound upon initial visit and, after undergoing laser therapy, it dropped to 33.0%. Therefore, there was a 54.1% reduction in the cases of patients with the cracking sounds (Graph 1).

The mean value of the initial mouth opening range of motion of the volunteers in our study was 41mm, while the final, measured one week after using the laser was 42.28mm, which represented an improvement of 1.28mm (3.12%). According to the Wilcoxon test it was statistically significant (p= 0.0409). The initial and final right side laterality were: 7.44mm and 7.6mm respectively, while those for the left wide were: 7.12mm and 7.88mm (Table 3).

**Table 3:** Values in millimeters of the mandible movements from the individuals in the experimental group.

Individuals	Mouth opening		Right side laterality		Left side laterality	
	Initial	Final	Initial	Final	Initial	Final
1	42	42	8	8	4	6
2	50	50	10	10	10	10
3	43	42	7	10	8	7
4	30	38	12	12	10	9
5	28	30	6	7	5	5
6	52	52	10	10	7	10
7	44	45	7	8	6	6
8	40	37	4	5	4	7
9	44	45	7	10	7	10
10	48	50	6	9	5	6
11	33	33	5	5	7	6
12	41	42	5	6	7	7
13	41	43	3	4	5	6
14	33	33	6	6	8	8
15	45	42	10	8	12	12
16	38	40	6	6	5	6
17	43	42	8	7	10	10
18	42	41	8	8	9	8
19	40	40	8	8	3	4
20	50	45	8	10	5	10
21	52	53	9	10	8	8
22	45	45	5	5	12	11
23	41	42	7	8	7	8
24	19	23	8	10	7	9
25	41	42	7	7	7	8
Mean	41,0	42,2	7,4	7,6	7,1	7,8
p-Value	0,0409*					

* Wilcoxon Test

## DISCUSSION

Despite the recent growing use of laser therapy, its action on the tissues is still inaccurate. It is known that, according to wavelength, the laser light has the capacity to alter cell functions (production of beta-endorphins, increase in protein synthesis, excretion, metabolism, cell division and repair) and, depending on the dose, it can inhibit or stimulate some of these functions^11^, ^12^.

Despite the lack of scientific evidence regarding its mechanism of action^10^, ^11^, some theories try to explain its therapeutic effects: greater release of beta-endorphins^11^; maintaining the nerve cell membrane potential – reducing the transmission of nerve signals^19^ and COX inhibition, reducing local pro-inflammatory substances^20^, and these all yield analgesic and anti-inflammatory effects.

Lopez^21^ stressed that, although laser therapy can reduce the pain in the individuals studied, in cases of muscle pain it returned shortly after to its initial values; nonetheless, in cases of joint pain, there was a marked reduction. Gray et al.^8^ described the laser as being efficient to control pain; however, they did not define it as the best treatment choice when compared to occlusal plaques, considered the best treatment option by the authors.

Placebo studies are paramount as long as ethics are respected. Refraining from using placebo can lead to the indication of inefficient treatment – to be considered as unethical behavior. Studies have shown that placebo, in fact, causes a biological response, as well as a behavioral one in a broad variety of medical conditions^22^. The placebo laser has been used in clinical research in an attempt to understand the true benefit of the therapeutic laser, although the results are still controversial^23^,^24^.

In the present study, there was a 43.6% reduction in pain in the individuals of the study group, in the control group, the reduction was only of 21.3%. However, since there was only one laser application, this result can be largely attributed to the positive psychological effect of the laser treatment in accordance with the studies from Pinheiro et al.^11^,^12^ after all, the control group also suffered a reduction, although of a lesser effect. Nonetheless, the different results between the study group and the control group reinforce the therapeutic value of the laser therapy, although it is not possible to rule out the possibility of its strengthening by the psychological effect, as well as the spontaneous regression of some acute flare up. Other studies did not report on the analgesic effect of the low power laser in the orofacial pain, being different from the results attained^17^,^23^,^25^. Although Hansen and Thoroe^17^ and Conti^10^ found pain reduction through the VAPS, they did not find significant differences between the study group and the placebo group.

The 54.16% reduction in the cases of patients with cracking sounds is corroborated by the study of Lopez^21^, in which one of the findings after laser therapy was a reduction in joint noises. On the other hand, it disagreed from the findings by Kulekcioglu et al.^15^, since they did not find the laser effects on the sounds present in the study groups (Graph 1).

**Table 4:** Values in millimeters of the mandible movements from the individuals in the control group.

Individuals	Mouth opening		Right side laterality		Left side laterality	
	Initial	Final	Initial	Final	Initial	Final
1	35	35	10	10	8	7
2	45	47	7	7	7	8
3	43	41	8	9	6	7
4	32	35	10	9	10	10
5	41	40	6	7	5	5
6	46	48	8	8	9	7
7	36	38	10	11	10	10
8	26	28	6	5	5	6
9	42	42	3	5	6	6
10	43	43	4	4	6	7
11	49	48	10	10	8	9
12	39	42	5	6	6	5
13	41	42	5	7	4	7
14	41	41	3	4	5	7
15	41	42	8	10	10	9
16	37	39	10	11	11	10
17	23	26	7	8	8	8
18	35	38	7	9	9	8
19	43	40	9	6	7	6
20	40	42	7	6	6	8
21	40	41	5	7	7	9
22	44	45	8	7	9	8
23	26	27	6	8	8	7
24	32	35	8	9	8	6
25	41	42	7	7	6	4
Mean	38,4	39,4	7,0	7,6	7,3	7,3
p-value	0,0972*					

* Wilcoxon Test

The results from this study, as well as those from other studies showed that there was a greater mean mandible range of motion after laser administration^9^,^10^,^15^,^18^, indicating that laser therapy was a means of treatment which caused satisfactory effects on the parameters used. The small variation can be associated to the quantity administered, although Pizzo et al.^26^ and Fikackova et al.^27^ found similar results from the placebo group.

## CONCLUSIONS

TMJ and the masseter muscle are the sites most affected by pain in patients with TMJD. The masseter and sternocleidomastoid muscles are frequently the most painful in those with joint noise. Laser therapy caused a reduction in the pain symptom after administration, by its analgesic action or a placebo effect resulting from an increase in the mean value of mandible movements. We found similar results in the control group, representing the positive psychological effect of laser therapy in these patients.
